# Biomarkers of cell damage, neutrophil and macrophage activation associated with in-hospital mortality in geriatric COVID-19 patients

**DOI:** 10.1186/s12979-022-00315-7

**Published:** 2022-12-15

**Authors:** M. Cardelli, E. Pierpaoli, F. Marchegiani, F. Marcheselli, F. Piacenza, R. Giacconi, R. Recchioni, T. Casoli, P. Stripoli, M. Provinciali, G. Matacchione, A. Giuliani, D. Ramini, J. Sabbatinelli, M. Bonafè, M. Di Rosa, A. Cherubini, C. Di Pentima, F. Spannella, R. Antonicelli, A. R. Bonfigli, F. Olivieri, F. Lattanzio

**Affiliations:** 1 Advanced Technology Center for Aging Research, Scientific Technological Area, IRCCS INRCA, Ancona, Italy; 2 Center of Clinical Pathology and Innovative Therapy, IRCCS INRCA, Ancona, Italy; 3 Center for Neurobiology of Aging, Scientific Technological Area, IRCCS INRCA, Via Birarelli 8, 60121 Ancona, Italy; 4 Department of Clinical and Molecular Sciences, Università Politecnica delle Marche, Via Tronto 10/a, 60126 Ancona, Italy; 5 SOD Medicina di Laboratorio, Azienda Ospedaliero Universitaria Ospedali Riuniti, Ancona, Italy; 6 Department of Experimental, Diagnostic, and Specialty Medicine (DIMES), University of Bologna, Bologna, Italy; 7 Unit of Geriatric Pharmacoepidemiology and Biostatistics, IRCCS INRCA, Cosenza, Italy; 8 Geriatria, Accettazione geriatrica e Centro di Ricerca per l’invecchiamento, IRCCS INRCA, Ancona, Italy; 9 Internal Medicine and Geriatrics, IRCCS INRCA, Via della Montagnola 81, 60127 Ancona, Italy; 10 Cardiology Unit, IRCCS INRCA, 60129 Ancona, Italy; 11 Scientific Direction and Geriatric Unit, IRCCS INRCA, Ancona, Italy

**Keywords:** COVID-19, SARS-CoV-2, Neutrophil elastase, sCD163, Prognostic biomarker, Geriatric patients, in-hospital mortality, cfDNA, Alu

## Abstract

**Background:**

The risk for symptomatic COVID-19 requiring hospitalization is higher in the older population. The course of the disease in hospitalised older patients may show significant variation, from mild to severe illness, ultimately leading to death in the most critical cases. The analysis of circulating biomolecules involved in mechanisms of inflammation, cell damage and innate immunity could lead to identify new biomarkers of COVID-19 severity, aimed to improve the clinical management of subjects at higher risk of severe outcomes.

In a cohort of COVID-19 geriatric patients (*n*= 156) who required hospitalization we analysed, on-admission, a series of circulating biomarkers related to neutrophil activation (neutrophil elastase, LL-37), macrophage activation (sCD163) and cell damage (nuclear cfDNA, mithocondrial cfDNA and nuclear cfDNA integrity). The above reported biomarkers were tested for their association with in-hospital mortality and with clinical, inflammatory and routine hematological parameters. Aim of the study was to unravel prognostic parameters for risk stratification of COVID-19 patients.

**Results:**

Lower n-cfDNA integrity, higher neutrophil elastase and higher sCD163 levels were significantly associated with an increased risk of in-hospital decease. Median (IQR) values observed in discharged vs. deceased patients were: 0.50 (0.30-0.72) vs. 0.33 (0.22-0.62) for n-cfDNA integrity; 94.0 (47.7-154.0) ng/ml vs. 115.7 (84.2-212.7) ng/ml for neutrophil elastase; 614.0 (370.0-821.0) ng/ml vs. 787.0 (560.0-1304.0) ng/ml for sCD163. The analysis of survival curves in patients stratified for tertiles of each biomarker showed that patients with n-cfDNA integrity < 0.32 or sCD163 in the range 492-811 ng/ml had higher risk of in-hospital decease than, respectively, patients with higher n-cfDNA integrity or lower sCD163. These associations were further confirmed in multivariate models adjusted for age, sex and outcome-related clinical variables. In these models also high levels of neutrophil elastase (>150 ng/ml) appeared to be independent predictor of in-hospital death. An additional analysis of neutrophil elastase in patients stratified for n-cfDNA integrity levels was conducted to better describe the association of the studied parameters with the outcome.

**Conclusions:**

On the whole, biomarkers of cell-free DNA integrity, neutrophil and macrophage activation might provide a valuable contribution to identify geriatric patients with high risk of COVID-19 in-hospital mortality.

**Supplementary Information:**

The online version contains supplementary material available at 10.1186/s12979-022-00315-7.

## Introduction

The coronavirus disease 2019 (COVID-19) global pandemic caused by the SARS coronavirus 2 (SARS-CoV-2) continues to pose significant challenges to global safety in public health. The pandemic, declared by the World Health Organization (WHO) on March 11, 2020, caused 375.465.073 total cases and 6.110.611 fatalities worldwide, as of 25/03/2022 [1]. Older patients with COVID-19, in particular, present higher risk of death compared to younger age groups. However, the severity of the disease and the clinical trajectories of the older infected cases show significant variation, from mild illness to severe disease requiring hospitalization and, in the most critic cases, the admission to intensive care unit [2]. This variability suggests the importance to find biomarkers of COVID-19 severity that can be used as personalised predictors, especially in the setting of older patients.

An exacerbate inflammatory response to severe acute respiratory syndrome coronavirus-2 (SARS-CoV-2) with the overproduction of many inflammatory cytokines, known as “cytokine storm” and often displaying the features of macrophage activation syndrome (MAS), is thought to be a major cause of disease severity and death in patients with COVID-19 pneumonia [3, 4]. Among the circulating biomolecules useful to unravel prognostic parameters for risk stratification of COVID-19 patients with the most severe disease, a number of biomarkers of neutrophil and macrophage activation were proposed [5]. Soluble (s)CD163 is the shed form of the corresponding membrane marker of monocyte/macrophage. As a result of the shedding, during inflammation and macrophage activation, the extracellular portion of CD163 circulates in the blood as sCD163 and its levels significantly increase during inflammatory responses, including SARS-CoV-2 infection [6]. In recent years, the CD163 receptor has also been reported to directly bind some pathogenic bacteria and also viruses [7].

Neutrophil elastase (NE), a serine proteases, and extracellular DNA are biomarkers of neutrophil extracellular traps (NET) formation [8]. The release of NETs, i.e. complexes created by cationic effectors, histones, and decondensed nuclear DNA (e.g., after histone citrullination), has been identified as one of the effector mechanisms utilized by neutrophils to kill microbes [9, 10]. Increasing evidence suggested that NETs can contribute to the inflammatory storm that leads to respiratory failure in many patients with COVID-19 [11–13].

In NETs, chromatin fibers are associated to chromatin proteins and other proteins released by neutrophils, including, among others, NE and cathelicidin (LL-37) [14]. The release of NETs, that is, NETosis, plays a major role in entrapping and eliminating pathogens, thus preventing their dissemination, including viruses, as confirmed for influenza [15, 16]. However, the beneficial role of NETs in the innate immune response coexists with its detrimental effects, such as the promotion of tissue damage, thrombosis, and autoimmunity [17, 18]. The inhibition of neutrophils and NETs was proven protective in various models of influenza-associated Acute Respiratory Distress Syndrome (ARDS) [19, 20]. Cell-free DNA (cfDNA) is present at low concentration in plasma in healthy conditions [21, 22]. Circulating cell-free DNA (cfDNA) mostly derives from cells that underwent apoptosis or necrosis and from the formation of NETs [23–25]. The relative contribution of the different processes by which cells release their nuclear DNA into body fluids can vary among different individuals and disease conditions, and this variability affects the value of cfDNA integrity, i.e. the ratio of longer to shorter DNA fragments in cfDNA [26]. Recent findings showed that in COVID-19, plasma cfDNA amount correlates with the WHO ordinal scale for disease progression [27]. Finally, cfDNA can be considered a powerful modulator of systemic inflammation, especially in older patients affected by COVID-19 [28].

In this work we aimed to evaluate the clinical significance and interlink between parameters of cell damages (n-cfDNA, mt-cfDNA, n-cfDNA integrity), neutrophil (NE and LL-37) and macrophage (sCD163) activation, in order to assess their prognostic value for risk stratification of COVID-19 patients with the most severe disease. Considering a cohort of geriatric COVID-19 patients admitted at the INRCA Hospital (Ancona), we analysed plasma/serum samples collected on admission, and verified the association of the selected biomarkers with survival and with clinical parameters.

## Results

### Clinical and laboratory characteristics of the patients in relation with in-hospital mortality

The clinical characteristics of the enrolled COVID-19 patients, grouped according to the outcome of hospital stay, are reported in Table 1. Patients deceased during hospital stay had higher median age and increased prevalence of stroke, chronic obstructive pulmonary disease (COPD) and chronic kidney disease (CKD). In addition, a significantly higher proportion of CFS (Clinical Frailty Scale) scores ≥ 8 was observed among deceased patients (Table 1). As expected, COVID-19 patients who died in hospital also had, at the admission, increased number of white blood cells (WBC), neutrophil % and neutrophil to lymphocyte ratio (NLR) and decreased counts of the other white blood cells. Among inflammatory factors and cytokines, CRP, IL-6, and IL-10 were significantly higher in patients who deceased during hospital stay. The proportion of plasma samples positive for SARS-CoV-2 RNA was about five-fold higher in patients who deceased compared to patients who survived (21/49 vs. 9/107, *p*<0.001).

**Table 1:** Description of clinical and laboratory characteristics of the patients, and differences based on the outcome

	**Total *N*=156**	**Discharged *N*=107**	**Deceased *N*=49**	***p*-value**
(A) **Patients’ characteristics and comorbidities**				
Age, median (IQR)	86 (82-90)	84 (81-89)	90 (85-93)	**<0.001**
Days of clinic stay, median (IQR)	13 (9-22)	14 (11-22)	10 (7-19)	**0.021**
Sex F, n (%)	97 (62.2%)	66 (61.7%)	31 (63.3%)	0.850
Hypertension, n (%)	109 (69.9%)	75 (70.1%)	34 (69.4%)	0.940
Diabetes, n (%)	37 (23.7%)	28 (26.2%)	9 (18.4%)	0.289
Stroke, n (%)	16 (10.3%)	7 (6.5%)	9 (18.4%)	**0.023**
Cancer, n (%)	37 (23.7%)	28 (26.2%)	9 (18.4%)	0.289
COPD, n (%)	22 (14.1%)	11 (10.3%)	11 (22.4%)	**0.042**
Asthma, n (%)	4 (2.6%)	4 (3.7%)	0 (0%)	0.171
Angina, n (%)	5 (3.2%)	4 (3.7%)	1 (2%)	0.577
Myocardial infarction, n (%)	18 (11.5%)	11 (10.3%)	7 (14.3%)	0.464
Atrial fibrillation, n (%)	45 (28.8%)	28 (26.2%)	17 (34.7%)	0.270
Hearth failure, n (%)	42 (26.9%)	25 (23.4%)	17 (34.7%)	0.135
Alzheimer, n (%)	16 (10.3%)	11 (10.3%)	5 (10.2%)	0.991
Dementia, n (%)	46 (29.5%)	32 (29.9%)	14 (28.6%)	0.870
CKD, Chronic Kidney Disease n (%)	38 (24.4%)	21 (19.6%)	17 (34.7%)	**0.041**
CFS, n (%)				**0.010**
0-3	25 (16%)	23 (21.5%)	2 (4.1%)	
4-7	77 (49.4%)	53 (49.5%)	24 (49%)	
8-9	52 (33.3%)	29 (27.1%)	23 (46.9%)	
NA	2 (1.3%)	2 (1.9%)	0 (0%)	
(B) **Routine laboratory parameters**				
WBC *10^3^, median (IQR)	8.5 (5.4-11.5)	7.9 (5.2-10.4)	11.8 (7.1-16.0)	**<0.001**
Neutrophils %, median (IQR)	79.2 (70.9-86.5)	75.9 (69.8-83.0)	87.7 (79.0-91.9)	**<0.001**
Neutrophil*10^3^, median (IQR)	6.0 (4.3-9.3)	5.5 (4.2-7.7)	9.5 (5.8-13.8)	**<0.001**
Lymphocytes %, median (IQR)	14.0 (8.6-22.6)	15.7 (11.0-24.2)	8.5 (4.1-14.0)	**<0.001**
Lymphocytes*10^3^, median (IQR)	1.1 (0.8-1.7)	1.2 (0.8-1.7)	0.9 (0.5-1.2)	**0.006**
Monocytes %, median (IQR)	6.0 (3.5-8.2)	7.2 (4.7-8.4)	3.5 (2.2-5.2)	**<0.001**
Monocytes, median (IQR)	0.48 (0.32-0.64)	0.49 (0.36-0.64)	0.41 (0.26-0.61)	**0.045**
Eosinophils %, median (IQR)	0.2 (0-0.7)	0.2 (0-0.9)	0 (0-0.3)	**<0.001**
Eosinophils*10^3^, median (IQR)	0.02 (0-0.06)	0.03 (0-0.07)	0 (0-0.02)	**0.001**
Basophils %, median (IQR)	0.1 (0.0-0.2)	0.1 (0.1-0.2)	0.1 (0.0-0.2)	**0.014**
Basophils*10^3^, median (IQR)	0.01 (0.01-0.02)	0.01 (0.01-0.02)	0.01 (0.01-0.02)	0.290
NLR, median (IQR)	5.4 (3.3-9.7)	4.5 (3.0-7.6)	11.1 (5.4-21.9)	**<0.001**
dNLR (derived neutrophil to lymphocyte ratio), median (IQR)	2.0 (0.7-4.2)	1.9 (0.7-3.4)	3.6 (0.6-7.3)	0.069
PLR (platelet to lymphocyte ratio), median (IQR)	225.2 (136.7-324.0)	209.1 (141.2-291.1)	250.6 (135.2-405.1)	0.125
LMR (lymphocyte to monocyte ratio), median (IQR)	2.3 (1.5-3.6)	2.4 (1.8-3.6)	1.8 (1.2-3.5)	0.100
(C) **SARS-CoV-2 RNAemia**				
SARS-CoV-2 RNAemia (2 viral genes detected), n (%)	30 (19.2%)	9 (8.4%)	21 (42.9%)	**<0.001**
(D) **Markers of inflammation and coagulation**				
D-DIMER ng/ml, median (IQR)	1090 (690-1890)	1030 (680-1690)	1260 (760-4190)	0.228
CRP mg/dL, median (IQR)	2.9 (1.1-8.0)	2.1 (0.7-6.1)	7.4 (2.4-12.7)	**<0.001**
IL-6 pg/ml, median (IQR)	59.7 (26.9-143.6)	46.9 (18.7-124.7)	99.0 (53.7-239.2)	**<0.001**
IL-10 pg/ml, median (IQR)	39.7 (25.7-60.8)	35.3 (24.5-55.7)	49.3 (37.1-89.9)	**<0.001**
IL-10/IL6 ratio, median (IQR)	0.68 (0.30-1.60)	0.73 (0.37-1.60)	0.52 (0.25-1.63)	0.161
TNF-α pg/ml, median (IQR)	0.92 (0.24-2.17)	1.02 (0.24-2.85)	0.69 (0.25-1.28)	0.055
IFNα pg/ml, median (IQR)	14.2 (3.6-47.3)	20.0 (5.0-44.7)	8.7 (0.0-47.3)	0.054
(E) **Patients’ drugs and treatments**				
Glucocorticoids, n (%)	133 (85.3%)	92 (85.2%)	41 (85.4%)	0.970
Heparin, n (%)	142 (91.0%)	96 (88.9%)	46 (95.8%)	0.161
Oxygen, n (%)				**0.006**
No	21 (13.5%)	21 (19.4%)	0 (0.0%)	
Standard oxygen	84 (53.8%)	57 (52.8%)	27 (56.3%)	
CPAP/High Flow	2 (1.3%)	2 (1.8%)	0 (0.0%)	
NIV	48 (30.8%)	27 (25.0%)	21 (43.7%)	
NA	1 (0.6%)	1 (1.0%)	0 (0.0%)	
(F) **Transfer to intensive care unit (ICU)**				
Transfer to intensive care unit (ICU), n (%)				0.691
No	150 (96.2%)	102 (95.3%)	48 (98.0 %)	
Yes	5 (3.2 %)	4 (3.7 %)	1 (2.0 %)	
NA	1 (0.6%)	1 (0.9 %)	0 (0.0 %)	

Font in bold: significant *p*-value. *NA* data not available

### Association of markers of cell damage, neutrophil, and macrophage activation with in-hospital mortality, and comparison of n-cfDNA parameters between infected and uninfected patients

As shown in Table 2, patients who deceased in-hospital had lower n-cfDNA integrity in plasma and higher levels of plasma NE and serum sCD163 with respect to discharged patients. Other tested biomarkers, including plasma concentration of n-cfDNA (Alu 247, Alu 115), mt-cfDNA and LL-37 were not significantly different between deceased and discharged patients. A ROC curve analysis found the following AUC (area under curve) (±std.err.) and *p*-values for the three biomarkers significantly associated with in-hospital death: for Alu 247/115, (n-cfDNA integrity) 0.6082 (±0.05), *p*= 0.033; for NE, 0.6409 (±0.046), *p* = 0.002; for sCD163, 0.6457 (±0.0491), *p*=0.003.

**Table 2:** Markers of cell damage, neutrophil, and macrophage activation in patients with different outcome

	Total (*N*=156)	Discharged (*N*=107)	Deceased (*N*=49)	*p*-value
Alu 115 (n-cfDNA ) pg/μl^a^, median(IQR)	361.7 (154.2-1041.9)	340.3 (163.9-1036.3)	385.2 (145.5-1109.1)	0.953
Alu 247 (n-cfDNA) pg/μl^a^, median(IQR)	136.9 (52.6-519.8)	163.4 (51.9-719.2)	109.7 (53.3-378.0)	0.449
Alu 247/115 (n-cfDNA integrity), median(IQR)	0.44 (0.33-0.77)	0.50 (0.30-0.72)	0.33 (0.22-0.62)	**0.031**
MT-CO3 (mt-cfDNA ) pg/μl^b^, median(IQR)	2.38 (1.12-5.22)	2.09 (1.00-5.13)	2.52 (1.48-6.23)	0.571
NE ng/ml , median(IQR)	105.1 (56.5-173.0)	94.0 (47.7-154.0)	115.7 (84.2-212.7)	**0.009**
LL-37 ng/mL), median(IQR)	39.0 (26.8-55.6)	36.9 (26.5-53.0)	44.3 (31.8-58.0)	0.194
sCD163 ng/mL median(IQR)	647.6 (441.0-951.9)	614.0 (370.0-821.0)	787.0 (560.0-1304.0)	**0.002**

^a^Absolute equivalent amount of genomic DNA/μl in the extracted sample. ^b^Absolute equivalent amount of the template (mitochondrial) DNA in the extracted sample. Font in bold: significant *p*-value

A comparison of n-cfDNA parameters in non-COVID-19 patients (Supplementary Tables S1 and S2) with those observed in COVID-19 patients found significantly higher values for all the three parameters (Alu 247, Alu 115, Alu 247/115) in COVID-19 patients (considered as a whole, or as subgroups of “discharged” or “deceased” patients) with respect to non-COVID-19 patients, with the partial exception of the Alu 247/115 parameter (not significantly different between non-COVID-19 and deceased COVID-19 patients).

### Distribution of plasma concentration of NE in patients with different outcomes and different levels of cfDNA integrity

**Fig. 1:**
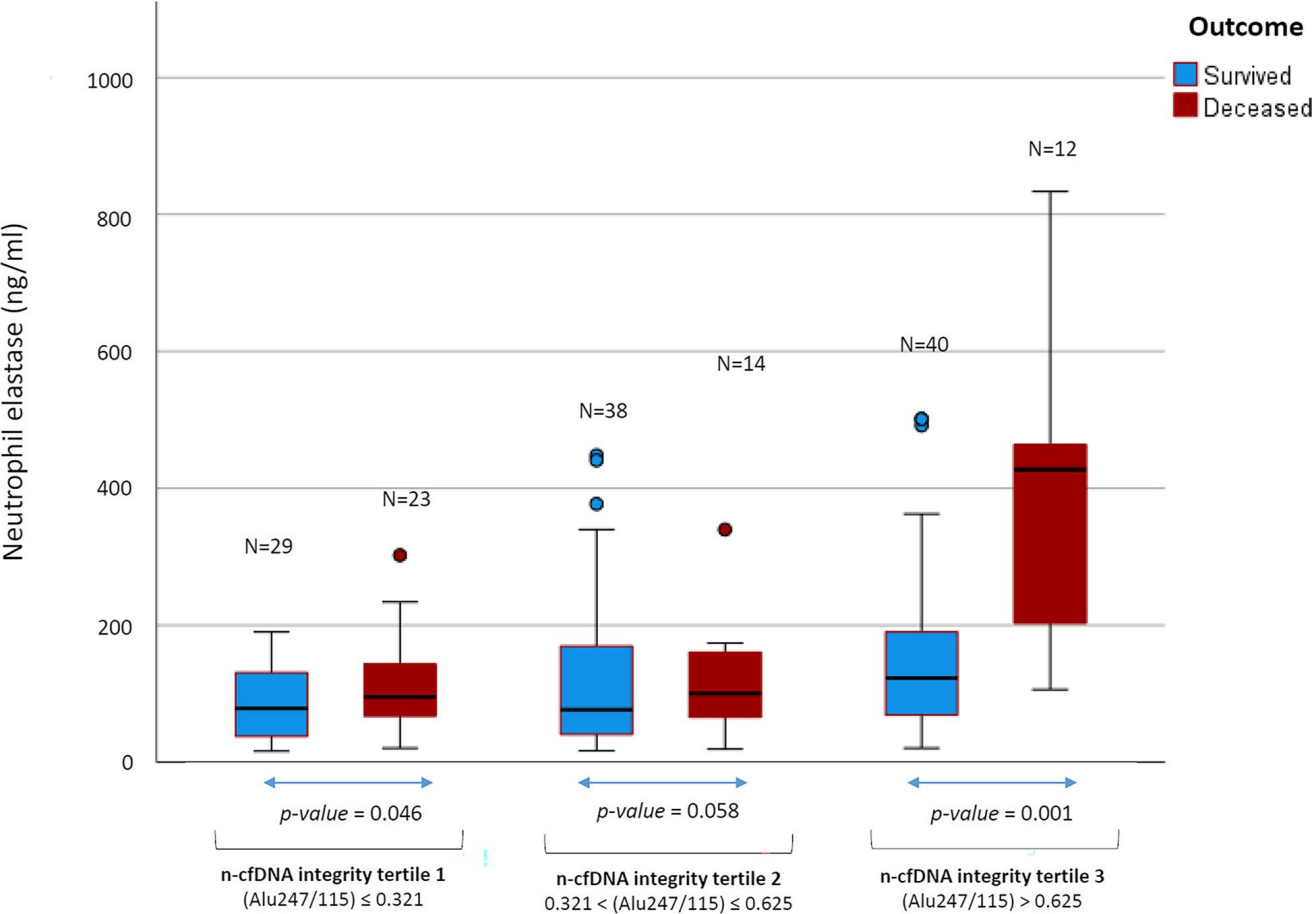
Distribution of plasma concentration of NE in patients with different outcomes and different levels of n-cfDNA integrity. The boxplots are grouped for different tertiles of n-cfDNA integrity (Alu 247/115). The significance of the different distribution of NE between deceased and survived patients in the same tertile of n-cfDNA integrity is shown under the graph

In order to further evaluate the relationship between the studied biomarkers and the outcome, the association of the NE level with the outcome was tested after stratifying for different values of cfDNA integrity (Fig. 1). Notably, in patients with high cfDNA integrity (Alu247/115 > 0.625, tertile 3), median concentration of NE was nearly three times higher in deceased compared to survived patients [median NE (IQR): 347.9 (203.1-463.9) ng/mL vs. 123.1 (59.6-190.4) ng/mL, *p*=0.001]. A ROC curve analysis conducted using the same kind of patients’ stratification (based on tertiles of cfDNA integrity) showed that the predictive value of NE for in-hospital death was higher for patients with highest n-cfDNA integrity (AUC ± std.err. for NE, in patients with n-cfDNA integrity in tertile 3, was 0.8354 ± 0.0623, *p*<0.001) than for patients with lower cf-DNA integrity (AUC ± std.err. 0.6627 ± 0.0758, *p*=0.032 or 0.5838 ± 0.0859, *p*=0.329, for NE in patients with n-cfDNA integrity in tertile 1 or in tertile 2 respectively).

### Association of markers of cell damage, neutrophil and macrophage activation with SARS-CoV-2 RNAemia

Table 3 shows that patients with detectable presence of SARS-COV-2 RNA in plasma had an increased abundance of n-cfDNA (Alu115), mt-cfDNA and NE. Other parameters, including integrity index of n-cfDNA (Alu247/115), sCD163 and LL-37 were not associated with SARS-CoV-2 RNAemia.

**Table 3:** Markers of cell damage, neutrophil and macrophage activation in COVID-19 patients with and without detectable SARS-CoV-2 RNAemia (Mann-Whitney independent samples test)

	Total	SARS-CoV-2 RNAemia (negative plasma samples, *n*=126)	SARS-CoV-2 RNAemia (positive plasma samples, *n*=30)	*p*-value
Alu 115 (n-cfDNA) pg/μl^a^, median(IQR)	361.67 (154.23-1041.85)	318.68 (145.52-904.82)	801.60 (382.90-1479.74)	**0.005**
Alu 247 (n-cfDNA) pg/μl^a^, median(IQR)	136.86 (52.62-519.81)	113.15 (46.52-417.98)	296.05 (74.86-931.76)	0.055
Alu 247/115 (n-cfDNA integrity), median(IQR)	0.44 (0.27-0.69)	0.46 (0.28-0.68)	0.32 (0.23-0.73)	0.380
MT-CO3 (mt-cfDNA ) pg/μl^**b**^, median(IQR)	2.380 (1.12-5.22)	1.985 (0.98-4.65)	4.26 (2.01-8.26)	**0.002**
NE ng/ml , median(IQR)	105.1 (56.50-172.95)	93.95 (48.60-154.50)	154.00 (100.20-234.00)	**<0.001**
LL-37 ng/mL, median(IQR)	39.0 (26.8-55.6)	39.3 (27.4-58.0)	34.05 (24.9-48.8)	0.501
sCD163, median(IQR)	647.60 (441.00-951.9000)	644.00 (375.00-940.45)	676.00 (541.80-1161.50)	0.149

^a^Absolute equivalent amount of genomic DNA/μl in the extracted sample. ^b^Absolute equivalent amount of the template (mitochondrial) DNA in the extracted sample. Font in bold: significant *p*-value

#### Evaluation of markers of cell damage, neutrophil, and macrophage activation as predictors of in-hospital survival

**Fig. 2:**
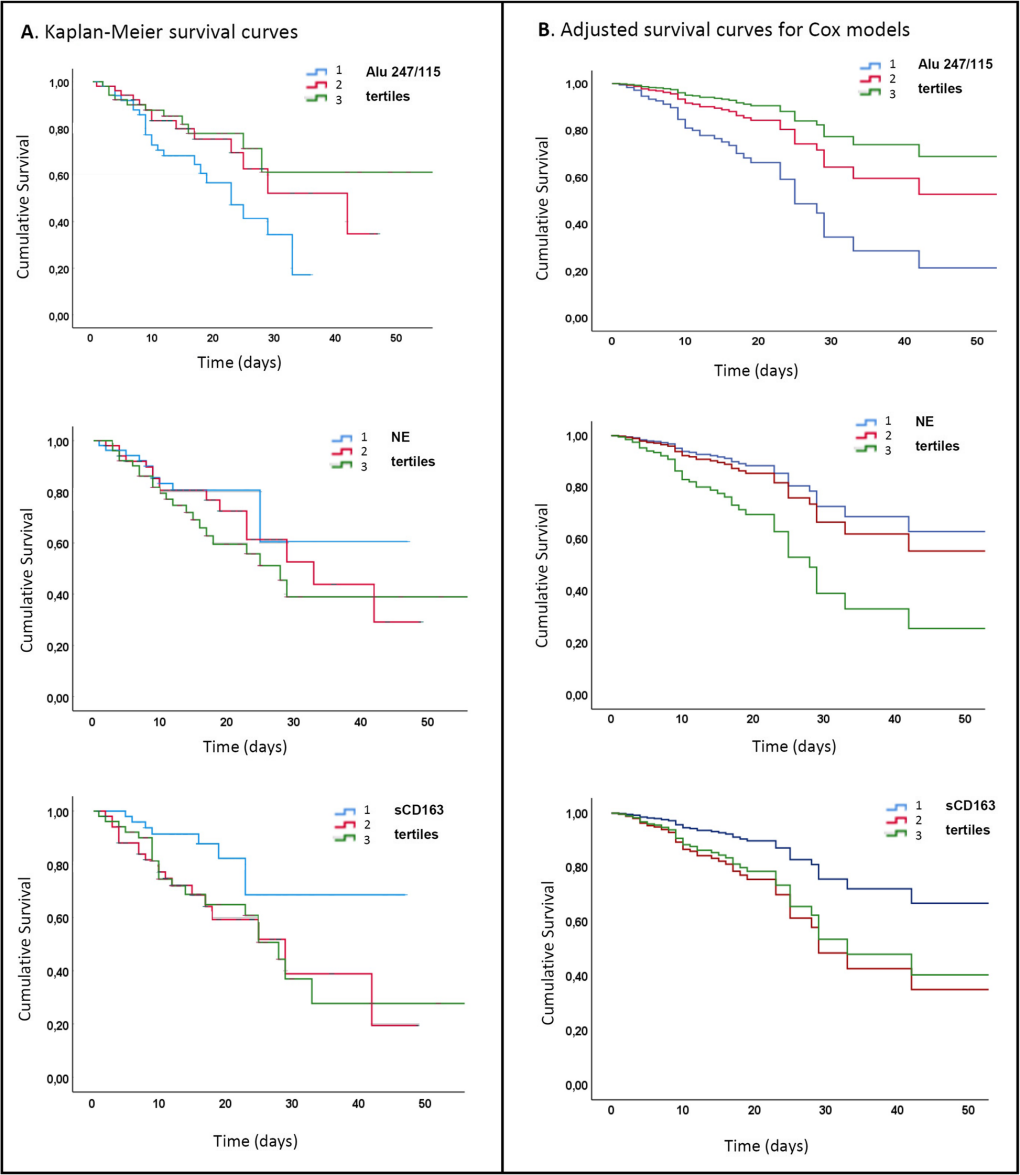
Survival curves survival of patients with different levels of n-cfDNA integrity, NE and sCD163. Each variable was categorized into tertiles as follows: for n-cfDNA, Alu247/115 ≤ 0.32 (tertile 1), 0.32 < Alu247/115 ≤ 0.62 (tertile 2), Alu247/115 > 0.62 (tertile 3); for NE, ≤ 74.0 ng/ml (tertile 1), 74 ng/ml < NE ≤ 146.7 ng/ml (tertile 2), NE > 146.7 ng/ml (tertile 3); for sCD163, ≤ 491 ng/ml (tertile 1), 491 ng/ml < sCD163 ≤ 811 ng/ml (tertile 2), sCD163 > 811 ng/ml (tertile 3). **A.** Upper graph: Kaplan-Meier survival estimates of patients with categorical values (tertiles) of n-cfDNA integrity index (Alu 247/115). Log-rank test for equality of survivor functions: p-value =0.039. Center: Kaplan-Meier survival estimates of patients with categorical values (tertiles) of NE. Log-rank test for equality of survivor functions: p-value = 0.441. Bottom: Kaplan-Meier survival estimates of patients with different categorical values (tertiles) of sCD163. Log-rank test for equality of survivor functions: p-value =0.020. **B.** Upper graph: Survival curves for different categorical values (tertiles) of cfDNA integrity index (Alu 247/115) adjusted for the covariates from the Cox proportional hazard “multivariate model 3”. Center: Survival curves for different categorical values (tertiles) of NE adjusted for the covariates from the Cox proportional hazard “multivariate model 3”. Bottom: Survival curves for different categorical values (tertiles) of sCD163 adjusted for the covariates from the Cox proportional hazard “multivariate model 3”

The three biomarkers showing differences between survived and deceased patients (Alu247/115, NE, sCD163) were then tested for their association with survival. To this purpose, each variable was categorized into tertiles. The Kaplan–Meier estimator was used as univariate analysis to estimate the overall survival associated with different levels of the three biomarkers. The results (Fig. 2A) show that patients with the lowest cfDNA integrity (Alu247/115 ≤ 0.32) have the lowest mean survival time compared to patients with average or higher n-cfDNA integrity: survival time (mean±sd) was 22.1 ± 1.9 days for tertile 1 (Alu247/115 ≤ 0.321); 32.2 ± 3.1 days for tertile 2 (0.321<Alu247/115≤0.625); 40.8 ± 4.3 days for tertile 3 (Alu247/115 > 0.625). On the other hand, patients with low levels of sCD163 (sCD163 < 491) show a longer survival with respect to the two subgroups with intermediate or high levels: 37.6 ± 3.0 days for tertile 1 (sCD163 ≤ 491 ng/ml); 27.3 ± 3.4 days for tertile 2 (491 ng/ml < sCD163 ≤ 811 ng/ml); 29.2 ± 3.8 days for tertile 3 (sCD163 > 811 ng/ml). When classifying patients according to NE, no significant differences were observed: survival time was 34.9±3.4 days for tertile 1 (NE ≤ 74.0 ng/ml); 31.3 ± 3.1 days for tertile 2 (74 ng/ml < NE ≤ 146.7 ng/ml); 31.6 ± 3.8 days for tertile 3 (NE > 146.7 ng/ml).

The three parameters (Alu 247/115, NE and sCD163) were also simultaneously tested for their association with survival in multivariate Cox regression models (Table 4) including sex and age (model 1), or sex, age, comorbidity and frailty (model 2), or all the previous variables and SARS-CoV-2 RNAemia (model 3). cfDNA integrity (Alu 247/115) was associated with the outcome in all the tested multivariate models. In particular, a lower cfDNA integrity index was significantly associated to an increased risk of in-hospital death in the three multivariate models. Higher levels of NE were associated with a higher risk of in-hospital death in multivariate models 2 and 3 (Table 4). Concerning sCD163, plasma concentration in the second tertile was associated with a higher risk of death in all the tested multivariate models (Table 4). The three tested biomarkers maintained their significant association with the outcome if pharmacological treatments were added to the multivariate Cox regression model, while two of them (Alu 247/115 and NE) maintained their significance even when oxygen therapy/ventilation was included as an additional confounding variable (Supplementary Table S3). A graphical representation of the adjusted survival curves for the three different biomarkers, based on the Cox multivariate model 3, is shown in Fig. 2 B.

**Table 4:** Cox proportional hazard ratios (HR) for survival based on different multivariate models

	Multivariate model (1)		Multivariate model (2)		Multivariate model (3)	
	HR (95%CI)	*p*-value	HR (95%CI)	*p*-value	HR (95%CI)	*p*-value
**n-cfDNA integrity tertiles**						
3 (Alu247/115 > 0.625)	1		1		1	
2 (0.321 < Alu247/115 ≤ 0.625)	1.55 (0.64-3.77)	0.332	1.56 (0.64-3.79)	0.328	1.72 (0.70-4.24)	0.241
1 (Alu247/115 < 0.321)	3.74 (1.61-8.74)	**0.002**	4.79 (2.00-11.48)	**<0.001**	4.14 (1.71-10.02)	**0.002**
**Neutrophil elastase tertiles**						
1 (NE ≤ 74.0)	1		1		1	
2 (74 < NE ≤ 146.7)	0.87 (0.39-1.96)	0.734	1.47 (0.61-3.50)	0.390	1.27 (0.52-3.11)	0.594
3 (NE > 146.7)	1.77 (0.78-3.99)	0.171	3.88 (1.53-9.86)	**0.004**	2.94 (1.10-7.89)	**0.032**
**sCD163 tertiles**						
1 (sCD163 ≤ 491)	1		1		1	
2 (491 < sCD163 ≤ 811)	3.06 (1.32-7.10)	**0.009**	2.78 (1.15-6.72)	**0.023**	2.60 (1.09-6.24)	**0.032**
3 (sCD163 > 811)	2.34 (0.99-5.50)	0.052	2.40 (0.98-5.89)	0.056	2.25 (0.91-5.52)	0.078
**Sex**						
Male	1		1		1	
Female	1.11 (0.60-2.03)	0.739	1.20 (0.63-2.28)	0.587	1.17 (0.61-2.23)	0.637
**Age**	1.10 (1.04-1.16)	**0.001**	1.09 (1.03-1.15)	**0.004**	1.08 (1.02-1.15)	**0.008**
**Stroke**						
No			1		1	
Yes			1.51 (0.66-3.44)	0.329	1.44 (0.62-3.34)	0.398
**COPD**						
No			1		1	
Yes			4.76 (2.07-10.94)	**<0.001**	4.47 (1.90-10.48)	**0.001**
**CKD**						
No			1		1	
Yes			0.89 (0.44-1.81)	0.745	0.85 (0.41-1.74)	0.656
**CFS**						
Ref. Cat. (0-3)			1		1	
1 (4-7)			4.92 (1.10-22.01)	**0.037**	4.63 (1.04-20.65)	**0.044**
2 (8-9)			4.53 (0.98-20.93)	0.053	4.32 (0.94-19.77)	0.059
**SARS-CoV-2 RNAemia**						
Negative					1	
Positive					1.91 (1.00-3.65)	0.051

Font in bold: significant *p*-value

### Correlation of markers of cell damage, neutrophil and macrophage activation with other laboratory parameters

Spearman rank correlation was tested to explore reciprocal associations among different plasma biomarkers of cell damage, neutrophil and macrophage activation (supplementary Table S4) and between these markers and other laboratory parameters (supplementary Table S5). A positive correlation was observed between the plasma concentration of n-cfDNA (Alu 115, Alu 247) and mt-cfDNA (MT-CO3), while the n-cfDNA integrity (Alu 247/115) in plasma was positively correlated with both parameters of n-cfDNA concentration but not with mt-cfDNA plasma concentration (supplementary Table S4). NE was positively correlated with all the cfDNA parameters (Alu 115, Alu 247, mt-cfDNA and Alu 247/115), sCD163 was positively correlated with one of the parameters of n-cfDNA concentration (Alu 115) and with NE, while LL-37 was not correlated with any of the above cited parameters (supplementary Table S4). Scatterplots with the individual data points of the correlation between NE levels and n-cfDNA parameters are shown in Supplementary Figures.

When analysing the relationship between cytokines, or other inflammatory markers, and the studied biomarkers, it resulted that both parameters of n-cfDNA abundance were negatively correlated with serum IFN-α, and one of them (Alu 247) was also positively correlated with serum IL-6 (supplementary Table S5). mt-cfDNA in plasma was positively correlated with serum IL-10. NE was positively correlated with the tested inflammatory parameters with exception of serum IFN-α (negatively correlated), fibrinogen and TNF-α (not correlated). Finally, LL-37 and sCD163 were not significantly correlated to the serum levels of any of the considered inflammatory parameters and cytokines (supplementary Table S5).

## Discussion

We assessed markers of cell damage, neutrophil and macrophage activation in hospitalized COVID-19 geriatric patients, identifying three plasma/serum parameters, i.e., integrity of n-cfDNA, NE and sCD163 levels, as significantly associated with in-hospital death .

In particular, concentrations of NE and sCD163 on-hospital admission were higher in patients who deceased in-hospital, in accordance with previous observations that exuberant neutrophil and macrophage activation is often associated with and could contribute to COVID-19 severity [4, 12, 13]. In addition, a third parameter, i.e. a low n-cfDNA integrity, was found to be associated with increased risk of in-hospital mortality. Different values of n-cfDNA integrity are considered to be representative of different origins of cfDNA: a low integrity should indicate a prevalent apoptotic origin, whereas higher integrity can be interpreted as a marker of necrosis [26, 29] or, as we could suggest, of abundant ongoing NETosis. Indeed, in our cohort of geriatric COVID-19 patients, n-cfDNA integrity was positively correlated with plasma NE, a well-recognized biomarker of NETosis. To explain this correlation, we could consider that NETosis is characterized by the release of webs of nuclear (and mithocondrial) DNA [30] in absence of DNA fragmentation [31], hence it is conceivable that massive NETosis involves an abundant release of relatively intact cfDNA in plasma. However, with an apparent paradox, n-cfDNA integrity and NE showed reciprocally direct correlation but opposite association with the outcome in the studied cohort (in-hospital death being associated with increased NE and with decreased n-cfDNA integrity). An analysis of NE in patients with different outcome, conducted after stratifying for different levels of cfDNA integrity, showed that NE was associated with a negative outcome only (or more significantly) in patients with high cfDNA integrity, i.e. in the subgroup of patients at lower risk of death. Conversely, most of the patients who deceased in hospital had low cfDNA integrity, but NE levels not significantly different (or only marginally different) from patients who survived. A possible interpretation for such data is that a combined analysis of n-cfDNA integrity and NE could help in identifying two groups of patients both with relatively high risk of mortality, but with different burden of NETosis: 1) patients with massive NETosis, showing high levels of elastase and high cfDNA integrity due to increased release of non-fragmented DNA from neutrophil; 2) patients without massive NETosis, showing levels of NE around or below the median value, and with low cfDNA integrity, possibly indicating increased apoptosis of immune cells. Considering that literature data show massive NETosis and T lymphocyte apoptosis as frequent characteristics of COVID-19 most severe outcomes,[23, 32] the prevalence of one or the other pathogenetic mechanism in different geriatric patients could help explaining the results obtained in this study. However, some considerations suggest caution when interpreting the present results: 1) as a circulating biomarker of NETosis, NE is not completely specific, since it can be released in plasma by neutrophils also by degranulation [33]; 2) the method of analysis of n-cfDNA employed in this study is not aimed to ascertain if the cfDNA derives from neutrophils or from other cell types. Hence, our hypothesis of different pathogenic mechanisms contributing to circulating n-cfDNA in different COVID-19 patients should be checked in future studies, possibly including the analysis of very specific biomarkers of NETosis such as the plasma levels of citrullinated histone H3 [34] or circulating myeloperoxidase (MPO)-DNA complexes [35].

Univariate survival analysis confirmed the association of n-cfDNA integrity and sCD163 with survival while, of note, multivariate analysis showed that n-cfDNA integrity, NE and sCD163 independently predict the outcome even if major prognostic clinical variables (SARS-CoV-2 RNAemia, comorbidities, frailty score, age and sex) are taken in consideration as covariates. Significantly, at least two of the studied biomarkers (n-cfDNA integrity, NE) appear to maintain their association with survival also when different interventions and medications are included in the multivariate model as additional confounding variables. Overall, the tested survival models confirmed that a lower risk of death was associated with higher n-cfDNA integrity, lower levels of NE and lower levels of sCD163.

Furthermore, absolute values of both n-cfDNA (Alu 115) and mt-cfDNA were associated with SARS-CoV-2 RNAemia, and n-cfDNA was also negatively correlated with serum INF-alpha. Considering that on-admission SARS-CoV-2 RNAemia and low serum IFN-alpha are both established markers of COVID-19 severe cases, [36, 37] the above reported observations are in agreement with the association between cfDNA and COVID-19 severity shown in other studies [34, 38].

The significantly increased levels of the most relevant cytokines modulating the inflammatory processes, such as IL-6 and IL-10, further support the notion that increased systemic inflammatory conditions are associated with poorer COVID-19 outcomes, especially in the setting of geriatric patients [39]. The observed positive correlation between IL-6, n-cfDNA abundance (ALU 247) and NE in plasma is in agreement with the results of a recent study showing that circulating markers of NET formation are associated with biomarkers of inflammation [34]. Of, note, the same authors also reported a prognostic value of circulating markers of NET formation including plasma level of NE, cfDNA abundance and citrullinated histone H3, [34] which is confirmed, in our study, for NE. The different results obtained for the parameters of cfDNA abundance (represented in our study by Alu 115 and Alu 247 values, not significantly associated with prognosis) could be attributed to the use of different methods for cfDNA analysis, or to the different (age) selection of COVID-19 cases. In addition, the elevated plasma levels of NET markers found in COVID-19 patients when compared to uninfected controls in the above cited study [34], could be considered in overall agreement with our results of an increased abundance of n-cfDNA (Alu 115 and Alu 247 DNA) in infected versus uninfected geriatric patients. Concerning the other main biomarkers analysed in this study, the median plasma concentration of NE and sCD163 observed in our cohort of COVID-19 patients was respectively at least five times higher, and one-point-six times higher, than the values (16-21 ng/ml and 400 ng/ml for NE and sCD163, respectively) reported in recent publications for control groups of 60-70 years old subjects [34, 40, 41]. Our study has some limitations that need to be addressed, most notably its retrospective nature and single-center design. In addition, the method used to extract nucleic acids from plasma samples was not specifically designed to collect highly fragmented DNA, and this could have potentially lead to an overall overestimation of the cfDNA integrity index, albeit without bias between different samples.

## Conclusions

On the whole, our data show that a combined analysis of n-cfDNA integrity, NE and sCD163 on hospital admission appear to be capable to discriminate geriatric COVID-19 patients at high risk of in-hospital death. If future studies will confirm the present results, the development of standardized clinical laboratory tests for cfDNA integrity, NE and sCD163 could be advisable, in order to facilitate the possible implementation of these biomarkers in the clinical practice.

## Materials and methods

### Patients

The present study utilizes data and biological samples from the Report-Age COVID project, an observational study conducted at the Italian National Center on Aging (IRCCS INRCA), Italy. The aim of this study is to provide a deeper understanding of COVID-19 disease in older hospitalized patients (age>65 years). All the selected subjects were cases of COVID-19 as confirmed by the positive detection of SARS-CoV-2 RNA in nasal/oro-pharyngeal swabs using real-time reverse transcriptase-polymerase chain reaction assay. The study protocol has been approved by the Ethics Committee of the IRCCS INRCA hospital, Ancona, Italy (reference number CE-INRCA-20008) and registered under the ClinicalTrials.gov database (reference number NCT04348396). Clinical and epidemiological data of hospitalization were gathered in a retrospective manner and anonymized prior to release. All the patients enrolled in the Report-Age COVID received treatment from INRCA hospital from March 1^st^ 2020 to date. Among these patients, we selected 156 subjects from the database based on the availability of biological samples (serum and plasma at hospital admission). These selected patients were admitted to INRCA hospital between October 11th and December 31st 2020. None of them received the anti-COVID-19 vaccine. Most of the patients received corticosteroids during their hospital stays, without significant differences with respect to the outcome. Standard oxygen or nonivasive ventilation (NIV) were used for most of the patients, while few patients were transferred to an intensive care unit (ICU). The initial decision to admit or not a COVID 19 older patient to the ICU was taken after a comprehensive evaluation by a multidisciplinary team, composed by the anesthesiologist, the geriatrician, the cardiologist and the palliativist, as appropriate, which also involved the patient and the family, whenever possible.

In addition to the cohort of COVID-19 patients, and in order to compare values for n-cfDNA parameters in hospitalized older patients with and without SARS-CoV-2 infection, a group of 36 older adults was selected from the Report-Age project, a large-scale ongoing observational study on the health conditions of hospitalized older adults at INRCA Hospital (Trial Registration no. NCT01397682) [42]. The study protocol of the Report-Age project has been approved by the Ethics Committee of the IRCCS INRCA hospital, Ancona, Italy [42]. The control group was composed of older adults who accessed the INRCA hospital for common geriatric disorders in the period 2013-2017 (before the COVID-19 pandemic outbreak), with available biological (plasma) samples and clinical and follow-up data. Patients with evidence of infectious or acute respiratory diseases or with intra-hospital mortality, or with less than two-years of follow-up survival after hospital discharge, were excluded. The patients of this control non-COVID-19 group had comparable sex ratio, median age and prevalence of most of the common age-related diseases as the analysed COVID-19 cohort.

### Plasma/serum collection and Nucleic acid extraction

EDTA plasma tubes were gently inverted 8 times and centrifuged. Tubes were centrifuged at 2500 x g at 4°C for 15 minutes. After the centrifugation of blood EDTA tube, three layers were obtained (plasma, buffy coat and erythrocytes). About 2.5 ml of plasma were collected from the upper part of the plasma layer, and placed in a new vial (avoiding collecting plasma from the lower part of the plasma layer near the buffy coat). The collected plasma EDTA volume was then subjected to a second centrifugation, conducted for 8 minutes at 10.000 RCF. After the centrifugation, the upper 80% of the volume was carefully collected (avoiding collecting of the lower 20% volume, enriched in cell debris and platelets) and stored in aliquots of 400-500 microliters, to be immediately frozen at -80°C. Immediately after collection, serum tubes were gently inverted 8 times and left at room temperature for 60 minutes to allow clotting, and then centrifuged at 2500 x g at 4°C for 15 minutes. After centrifugation, the top of the supernatant was carefully aspirated and stored in aliquots of 500-1000 microliters, to be immediately frozen at -80°C. Nucleic acids were extracted from 140 μl of plasma using the QIAamp Viral RNA Mini Kit (Qiagen, Hilden, Germany). The kit does not discriminate between RNA and DNA, and it was used to extract plasma cell free DNA (of cellular origin) and viral RNA (for detection of SARS-CoV-2 RNAeamia) from the same plasma samples. The purified nucleic acid sample was conserved at -80°C before the analysis.

### mt-cfDNA quantification

PCR amplification of mitochondrial cfDNA (mt-cfDNA) was measured using a real-time quantitative assay for Human cytochrome C oxidase subunit III (MT-CO3) gene. All assays were performed on a Rotor-Gene Q detection system (Qiagen) using a 72-well carousel.

The reaction mixture consisted of 2 μl of nucleic acids from plasma samples and 18 μl master mix, which was composed of 7 μl H2O, 10 μl SYBR qPCR Master Mix (Vazyme, Nanjing, China) and 1 μl of 5 μM forward and reverse primers, respectively. The primers used were: forward 5’-ATGACCCACCAATCACATGC-3’, reverse 5’- ATCACATGGCTAGGCCGGAG-3’ (IDT, Coralville, IA).

PCR conditions were set to: 95 °C for 3 min, followed by 40 cycles of 10 s denaturation at 95 °C, 30 s annealing at 55 °C. Absolute quantification of the target sequence in each biological sample was estimated by comparison to a RT-qPCR standard amplification curve. To generate the standard MT-DNA, a selected region of human purified genomic DNA containing the target sequence for MT-CO3 (forward 5’-ATGACCCACCAATCACATGC-3’, reverse 5’-ATCAATAGATGGAGACATAC-3’) was amplified by PCR under the following conditions: initial denaturation at 95° C for 1 min, then 35 cycles of denaturation at 95° C for 30 s, annealing at 50° C for 30 s and extension at 72°C for 1 min. The generated amplicon had a length of 775 nucleotides and a molecular weight of 478879.57 Da. After amplification, the MT-DNA standard was purified by using MinElute columns (Qiagen) and quantified using a NanoDrop spectrophotometer (ThermoScientific). Serial dilutions were then used to calibrate the RT-qPCR standard curves. Each sample was quantified in duplicate, and triplicates of the standard curve were included in each run.

### Determination of abundance and integrity of n-cfDNA

The polymerase chain reaction protocol used for the quantification of nuclear cfDNA (n-cfDNA) and for the evaluation of n-cfDNA integrity was derived from a previous study [26]. Two sets of primers complementary to the consensus sequence of human Alu repeats were used: ALU115 primer forward, 5’-CCTGAGGTCAGGAGTTCGAG-3’; ALU115 primer reverse, 5’-CCCGAGTAGCTGGGATTACA-3’; ALU247 primer forward, 5’-GTGGCTCACGCCTGTAATC-3’; ALU247 primer reverse, 5’-CAGGCTGGAGTGCAGTGG-3’. The first set was used to amplify the 115-bp amplicon (ALU 115), the second for the 247-bp amplicon (ALU 247). The reaction was conducted using 0.2 μM each of forward primer and reverse primer (ALU 115 or ALU 247), 1x Ssofast mix containing enzyme and Syber Green dye (Bio-Rad Laboratories), in a reaction volume of 15 μl. Thermal cycles were conducted on a Qiagen Rotor-Gene instrument, as follows: pre-denaturation 2.5 min at 98°C, followed by 35 cycles of denaturation 15 sec at 95°, annealing/extension 60 sec at 64°C. A melting curve was conducted at the end of each run, to check for the presence of unspecific amplification. A calibration curve constructed by amplifying serial dilutions of a genomic DNA standard sample (0.02 to 200 pg/μl) was present in each assay run, and was used to assess the absolute equivalent concentration of genomic DNA in each plasma sample. Each sample was run in duplicate.

All assays were conducted in blind without knowing the sample identity. The ratio of concentrations calculated for each sample with the two set of primers, i.e. (concentration of Alu 247) / (concentration of Alu 115), hereafter indicated as Alu247/115, was used as index of n-cfDNA integrity.

### Measurement of soluble biomarkers

Serum concentration of IL-6, IFN-α, TNF-α and IL-10 were assessed by using four Pro Quantum Immunoassays (Thermo Fisher Scientific) specific for each of the four biomolecules. Each reaction was conducted in duplicate, using 2 μl of serum, and run on an Agilent Aria Mx Real Time PCR instrument at the conditions indicated by the producer.

The plasma levels of NE and LL37 were measured using commercial ELISA kits (ABCAM, AB119553 PMN Elastase Human ELISA Kit and Hycult Biotech, HK321 Human LL-37). The serum levels of CD163 were measured using a commercial ELISA kit (R&DSystems, Human CD163 Immunoassay). All the serum/plasma samples were diluted 1:20 (for sCD163 and LL37 assayes) or 1:50 (for neutrophile elastase assay). All samples were ran in duplicate and samples with an intra-assay coefficient of variation below 10.0% were included in this study.

### Assessment of RNAemia

The presence of SARS-CoV-2 RNA in plasma was assessed by analyzing 10 μl of nucleic acids extracted from plasma in a Real Quality RQ-SARS-CoV-2 real time PCR assay (AB Analitica). The assay was specific for the genes RdRp (encoding the RNA-dependent RNA polymerase) gene and S gene (encoding the spike protein). The presence of an endogenous control (targeting the human RNAse P gene) in the same assay was used as internal control to ensure proper sampling and nucleic acids extraction. The assay was used following the conditions indicated by the producer, on an Agilent Aria Mx Real Time PCR instrument. The plasma sample was considered positive for the SARS-CoV-2 RNAemia if both viral genes were detected with a cycle threshold (CT) < 40.

### Statistical analysis

Continuous variables were reported as median and interquartile range after their non-normality have been assessed using Shapiro-Wilk test and comparison of variables between groups was performed by unpaired Mann-Whitney U test. Tertiles of NE, sCD163 and n-cfDNA integrity (Alu247/115) were calculated and, as other categorical variables, were expressed as absolute number.

Spearman rank correlation was conducted to check associations among different biomarkers and between these biomarkers and other parameters.

The Kaplan–Meier survival curves were used to estimate the association of in-hospital mortality risk with different levels of the analysed biomarkers. Cox proportional hazards analysis were used to derive age- and gender-adjusted (Model 1); age- gender- comorbidities- and Clinical Frailty Scale (CFS)-adjusted (Model 2); age- gender- comorbidities- Clinical Frailty Scale (CFS)- and SARS-CoV-2 RNAemia-adjusted (Model 3) and age- gender- comorbidities- Clinical Frailty Scale (CFS)- SARS-CoV-2 RNAemia- and treatments-adjusted (Model 4 and Model 5) hazard ratios (HR) and 95% confidence intervals (95% CI) of the association between all independent variables and study outcome. The choice to consider different Cox proportional hazards models, each containing the same tested variables (biomarkers), but adjusted for an increasing number of confounders, was aimed to verify if a statistically significant relationship between a biomarker and survival maintained its significance after the addition of confounders, and which (if any) confounders could determine a loss of significance in the tested variables. The length of hospital stay was used as the time to failure variable for the model. A two-tailed P value < .05 was considered significant.

The statistical analyses were performed using the IBM SPSS Statistics program (version 27) and the STATA version15.1 Statistical Software Package for Windows (Stata Corp, College Station, TX).

## Supplementary Information

**Additional file 1:** **Supplementary Table S1.** Characteristics and comorbidities of non-COVID-19 patients.

**Additional file 2:** **Supplementary Table S2.** Parameters of n-cfDNA in non-COVID-19 geriatric patients and comparison with COVID-19 patients.

**Additional file 3:** **Supplementary Table S3.** Cox proportional hazard ratios (HR) for in-hospital survival of COVID-19 patients based on different multivariate models (with pharmacological treatments/oxygen therapy as additional confounding variables).

**Additional file 4:** **Supplementary Table S4.** Correlation among markers of cell damage, neutrophil and macrophage activation.

**Additional file 5:** **Supplementary Table S5.** Correlation of markers of cell damage, neutrophil and macrophage activation with other laboratory parameters.

**Additional file 6:** **Supplementary Figures.** Scatterplots with the individual data points of the correlation between NE levels and n-cfDNA parameters.

## Data Availability

The dataset obtained during the current study are part of a more extensive unpublished work, and thus are not publicly available. However, manuscript data can be made available from the corresponding author upon reasonable request.
