# Supplementary material for: Biomarkers of cell damage, neutrophil and macrophage activation associated with in-hospital mortality in geriatric COVID-19 patients
Source: Immun Ageing. 2022 Dec 15;19:65. doi: 10.1186/s12979-022-00315-7 (PMC9751505; doi:10.1186/s12979-022-00315-7)
Supplement: Supplementary file 1 — Additional file 1: Supplementary Table S1. Characteristics and comorbidities of non-COVID-19 patients. [file 12979_2022_315_MOESM1_ESM.docx]

**Supplementary Table S1.** *Characteristics and comorbidities of non-COVID-19 geriatric patients*

| **Characteristics and comorbidities** | **Total**  **n = 36** |
| --- | --- |
| Age, median(IQR) | 85 (84-88) |
| Sex F, n (%) | 21 (58.3%) |
| Hypertension, n (%) | 25 (69.4%) |
| Diabetes, n (%) | 8 (22.2%) |
| Stroke, n (%) | 1 (2.7%) |
| Cancer, n (%) | 6 (16.7%) |
| COPD, n (%) | 9 (25%) |
| Asthma, n (%) | 0 (0%) |
| Angina, n (%) | 0 (0%) |
| Myocardial infarction, n (%) | 1 (2.8%) |
| Atrial fibrillation, n (%) | 8 (22.2%) |
| Hearth failure, n (%) | 6 (16.7%) |
| Alzheimer, n (%) | 0 (0%) |
| Dementia, n (%) | 2 (5.6%) |
| CKD, Chronic Kidney Disease n (%) | 10 (27.8%) |
