# Supplementary material for: Biomarkers of cell damage, neutrophil and macrophage activation associated with in-hospital mortality in geriatric COVID-19 patients
Source: Immun Ageing. 2022 Dec 15;19:65. doi: 10.1186/s12979-022-00315-7 (PMC9751505; doi:10.1186/s12979-022-00315-7)
Supplement: Supplementary file 2 — Additional file 2: Supplementary Table S2. Parameters of n-cfDNA in non-COVID-19 geriatric patients and comparison with COVID-19 patients. [file 12979_2022_315_MOESM2_ESM.docx]

**Supplementary Table S2.** *Parameters of n-cfDNA in non-COVID-19 geriatric patients and comparison with COVID-19 patients*

|  | ***Non-COVID-19***  ***geriatric patients***  ***(n=36)*** | ***COVID-19***  ***geriatric patients***  ***(Total, n=156)*** | ***COVID-19***  ***geriatric patients***  ***(Discharged, n=107)*** | ***COVID-19***  ***geriatric patients***  ***(Deceased, n=49)*** |
| --- | --- | --- | --- | --- |
| Alu 115 (n-cfDNA ) pg/μl^#^, median(IQR) | 193.8  (114.7-309.1) | 361.7  (154.2-1041.9)  **^**^** | 340.3  (163.9-1036.3)  **^**^** | 385.2  (145.5-1109.1)  **^*^** |
| Alu 247 (n-cfDNA) pg/μl^#^, median(IQR) | 71.4  (46.2-108.9) | 136.9  (52.6-519.8)  **^**^** | 163.4  (51.9-719.2)  **^**^** | 109.7  (53.3-378.0)  **^*^** |
| Alu 247/115 (n-cfDNA integrity), median(IQR) | 0.335  (0.26-0.46) | 0.44  (0.33-0.77)  **^*^** | 0.50  (0.30-0.72)  **^**^** | 0.33  (0.22-0.62) |
|  |  |  |  |  |

^#^ *Absolute equivalent amount of genomic DNA/μl in the extracted sample.*

******* *p-value<0.05 (Mann-Whitney U test) when the variable is compared between the selected group and non-COVID-19 patients*

******** *p-value<0.01 (Mann-Whitney U test) when the variable is compared between the selected group and non-COVID-19 patients*
