# Supplementary material for: Biomarkers of cell damage, neutrophil and macrophage activation associated with in-hospital mortality in geriatric COVID-19 patients
Source: Immun Ageing. 2022 Dec 15;19:65. doi: 10.1186/s12979-022-00315-7 (PMC9751505; doi:10.1186/s12979-022-00315-7)
Supplement: Supplementary file 3 — Additional file 3: Supplementary Table S3. Cox proportional hazard ratios (HR) for in-hospital survival of COVID-19 patients based on different multivariate models (with pharmacological treatments/oxygen therapy as additional confounding variables). [file 12979_2022_315_MOESM3_ESM.docx]

**Supplementary Table S3.**

Cox proportional hazard ratios (HR) for survival based on different multivariate models (with pharmacological/oxygen therapy as additional confounding variables)

|  | **Multivariate model**  **(4)** | | **Multivariate model**  **(5)** | |
| --- | --- | --- | --- | --- |
|  | **HR (95%CI)** | **p-value** | **HR (95%CI)** | **p-value** |
| **n-cfDNA integrity tertiles** |  |  |  |  |
| 3 (Alu247/115 > 0.625) | 1 |  | 1 |  |
| 2 (0.321 < Alu247/115 ≤ 0.625) | 2.02 (0.79-5.17) | 0.143 | 1.84 (0.71-4.77) | 0.213 |
| 1 (Alu247/115 < 0.321) | 4.97 1.98-12.45) | **0.001** | 5.15 (2.06-12.89) | **<0.001** |
| **Neutrophil elastase tertiles** |  |  |  |  |
| 1 (NE ≤ 74.0) | 1 |  | 1 |  |
| 2 (74 < NE ≤ 146.7) | 1.37 (0.55-3.40) | 0.494 | 1.60 (0.62-4.10) | 0.329 |
| 3 (NE > 146.7) | 3.06 (1.14-8.22) | **0.026** | 3.91 (1.41-10.81) | **0.009** |
| **sCD163 tertiles** |  |  |  |  |
| 1 (sCD163 ≤ 491) | 1 |  | 1 |  |
| 2 (491 < sCD163 ≤ 811) | 2.65 (1.08-6.47) | **0.033** | 2.35 (0.95-5.79) | 0.063 |
| 3 (sCD163 > 811) | 2.43 (0.96-6.12) | 0.060 | 1.97 (0.78-4.98) | 0.150 |
| **Sex** |  |  |  |  |
| Male | 1 |  | 1 |  |
| Female | 1.11 (0.57-2.14) | 0.763 | 1.36 (0.67-2.76) | 0.393 |
| **Age** | 1.09 (1.03-1.16) | **0.004** | 1.09 (1.03-1.16) | **0.005** |
| **Stroke** |  |  |  |  |
| No | 1 |  | 1 |  |
| Yes | 1.51 (0.65-3.51) | 0.334 | 1.72 (0.71-4.16) | 0.233 |
| **COPD** |  |  |  |  |
| No | 1 |  | 1 |  |
| Yes | 4.73 (2.04-10.97) | **<0.001** | 5.67 (2.37-13.56) | **<0.001** |
| **CKD** |  |  |  |  |
| No | 1 |  | 1 |  |
| Yes | 0.90 (0.44-1.87) | 0.783 | 0.86 (0.40-1.85) | 0.707 |
| **CFS** |  |  |  |  |
| Ref. Cat. (0-3) | 1 |  | 1 |  |
| 1 (4-7) | 4.07 (0.90-18.47) | 0.069 | 4.28 (0.92-19.91) | 0.064 |
| 2 (8-9) | 3.76 (0.81-17.38) | 0.090 | 4.12 (0.83-20.41) | 0.083 |
| **SARS-CoV-2 RNAemia** |  |  |  |  |
| Negative | 1 |  | 1 |  |
| Positive | 1.70 (0.86-3.33) | 0.124 | 1.59 (0.77-3.27) | 0.210 |
| **Glucocorticoids** |  |  |  |  |
| No | 1 |  | 1 |  |
| Yes | 0.43 (0.17-1.09) | 0.076 | 0.47 (0.16-1.33) | 0.153 |
| **Heparin** |  |  |  |  |
| No | 1 |  | 1 |  |
| Yes | 1.86 (0.24-14.24) | 0.550 | 1.85 (0.24-14.39) | 0.558 |
| **Oxygen** |  |  |  |  |
| Ref. Cat. (none/standard oxygen) |  |  | 1 |  |
| CPAP/High Flow/NIV |  |  | 1.19 (0.57-2.49) | 0.642 |
