# Supplementary material for: Biomarkers of cell damage, neutrophil and macrophage activation associated with in-hospital mortality in geriatric COVID-19 patients
Source: Immun Ageing. 2022 Dec 15;19:65. doi: 10.1186/s12979-022-00315-7 (PMC9751505; doi:10.1186/s12979-022-00315-7)
Supplement: Supplementary file 4 — Additional file 4: Supplementary Table S4. Correlation among markers of cell damage, neutrophil and macrophage activation. [file 12979_2022_315_MOESM4_ESM.docx]

**Supplementary Table S4.** *Spearman rank correlation coefficients and p-values between different markers of cell damage, neutrophil and macrophage activation.*

|  | **n-cfDNA (Alu 115)** | **n-cfDNA (Alu 247)** | **n-cfDNA integrity (Alu 247/115)** | **mt-cfDNA (MT-CO3)** | **Neutrophil Elastase** | **LL37** | **sCD163** |
| --- | --- | --- | --- | --- | --- | --- | --- |
| **n-cfDNA (Alu 115)** | 1 |  |  |  |  |  |  |
| **n-cfDNA (Alu 247)** | **0.9244**  (p<0.001) | 1 |  |  |  |  |  |
| **n-cfDNA integrity**  **(Alu 247/115)** | **0.3563**  (p<0.001) | **0.6576**  (p<0.001) | 1 |  |  |  |  |
| **mt-cfDNA (MT-CO3)** | **0.5110**  (p<0.001) | **0.4254**  (p<0.001) | 0.0472  (p=0.569) | 1 |  |  |  |
| **Neutrophil Elastase (ng/ml)** | **0.5417**  (p<0.001) | **0.5377**  (p<0.001) | **0.2770**  (p=0.001) | **0.3457**  (p<0.001) | 1 |  |  |
| **LL-37 (ng/mL)** | 0.0004  (p=0.996) | 0.0596  (p=0.472) | 0.1128  (p=0.172) | 0.0082  (p=0.921) | 0.1375  (p=0.096) | 1 |  |
| **sCD163** | **0.1825**  (p=0.026) | 0.0836  (p=0.312) | -0.1049  (p=0.205) | 0.1448  (p=0.079) | **0.1662**  (p=0.044) | 0.017  (p=0.838) | 1 |

Values in bold: significant correlations.
