# Supplementary material for: Biomarkers of cell damage, neutrophil and macrophage activation associated with in-hospital mortality in geriatric COVID-19 patients
Source: Immun Ageing. 2022 Dec 15;19:65. doi: 10.1186/s12979-022-00315-7 (PMC9751505; doi:10.1186/s12979-022-00315-7)
Supplement: Supplementary file 5 — Additional file 5: Supplementary Table S5. Correlation of markers of cell damage, neutrophil and macrophage activation with other laboratory parameters. [file 12979_2022_315_MOESM5_ESM.docx]

**Supplementary Table S5.** *Spearman rank correlation coefficients and p-values between markers of cell damage, neutrophil and macrophage activation and other laboratory parameters.*

|  | **n-cfDNA (Alu 115)** | **n-cfDNA (Alu 247)** | **n-cfDNA integrity (Alu 247/115)** | **mt-cfDNA (MT-CO3)** | **Neutrophil Elastase** | **LL37** | **sCD163** |
| --- | --- | --- | --- | --- | --- | --- | --- |
| **NLR** | 0.0710 (p=0.468) | 0.0242 (p=0.804) | -0.1286 (p=0.187) | 0.1245 (p=0.202) | **0.2025** (p=0.036) | 0.0898 (p=0.358) | 0.1566 (p=0.107) |
| **D dimer** | 0.0292 (p=0.765) | -0.0082 (p=0.934) | -0.0204 (p=0.835) | 0.0150 (p=0.878) | **0.2108** (p=0.029) | 0.1196 (p=0.220) | **0.2125** (p=0.028) |
| **Fibrinogen** | 0.0429 (p=0.661) | -0.0196 (p=0.841) | -0.1635 (p=0.093) | 0.1767 (p=0.069) | 0.0852 (p=0.383) | 0.1457 (p=0.134) | 0.0216 (p=0.825) |
| **CRP** | 0.1602 (p=0.099) | 0.1255 (p=0.198) | -0.0165 (p=0.866) | 0.1595 (p=0.101) | **0.2139** (p=0.027) | 0.0368 (p=0.707) | 0.1524 (p=0.117) |
| **IL6** | 0.1645 (p=0.091) | **0.2001** (p=0.039) | 0.1515 (p=0.119) | 0.1521 (p=0.118) | **0.2647** (p=0.006) | 0.1860 (p=0.055) | 0.1131 (p=0.246) |
| **IFN-alpha** | **-0.2004** (p=0.038) | **-0.2481** (p=0.010) | **-0.2331** (p=0.016) | -0.0766 (p=0.433) | **-0.2689** (p=0.005) | -0.1268 (p=0.193) | -0.0098 (p=0.920) |
| **Il10** | 0.1744 (p=0.073) | 0.0771 (p=0.430) | -0.1665 (p=0.087) | **0.3785** (p<0.001) | **0.3201** (p=0.001) | -0.027 (p=0.782) | **0.2162** (p=0.025) |
| **TNF-alpha** | -0.0925 (p=0.343) | 0.0050 (p=0.959) | **0.2164** (p=0.025) | -0.0998 (p=0.306) | -0.1309 (p=0.179) | 0.0929 (p=0.341) | -0.1026 (p=0.293) |

Values in bold: significant correlations.
