# Supplementary material for: Biomarkers of cell damage, neutrophil and macrophage activation associated with in-hospital mortality in geriatric COVID-19 patients
Source: Immun Ageing. 2022 Dec 15;19:65. doi: 10.1186/s12979-022-00315-7 (PMC9751505; doi:10.1186/s12979-022-00315-7)

Supplemental figures.

Scatterplots with the individual data points of the correlation between NE levels and n-cfDNA (Alu 115 and Alu 247)

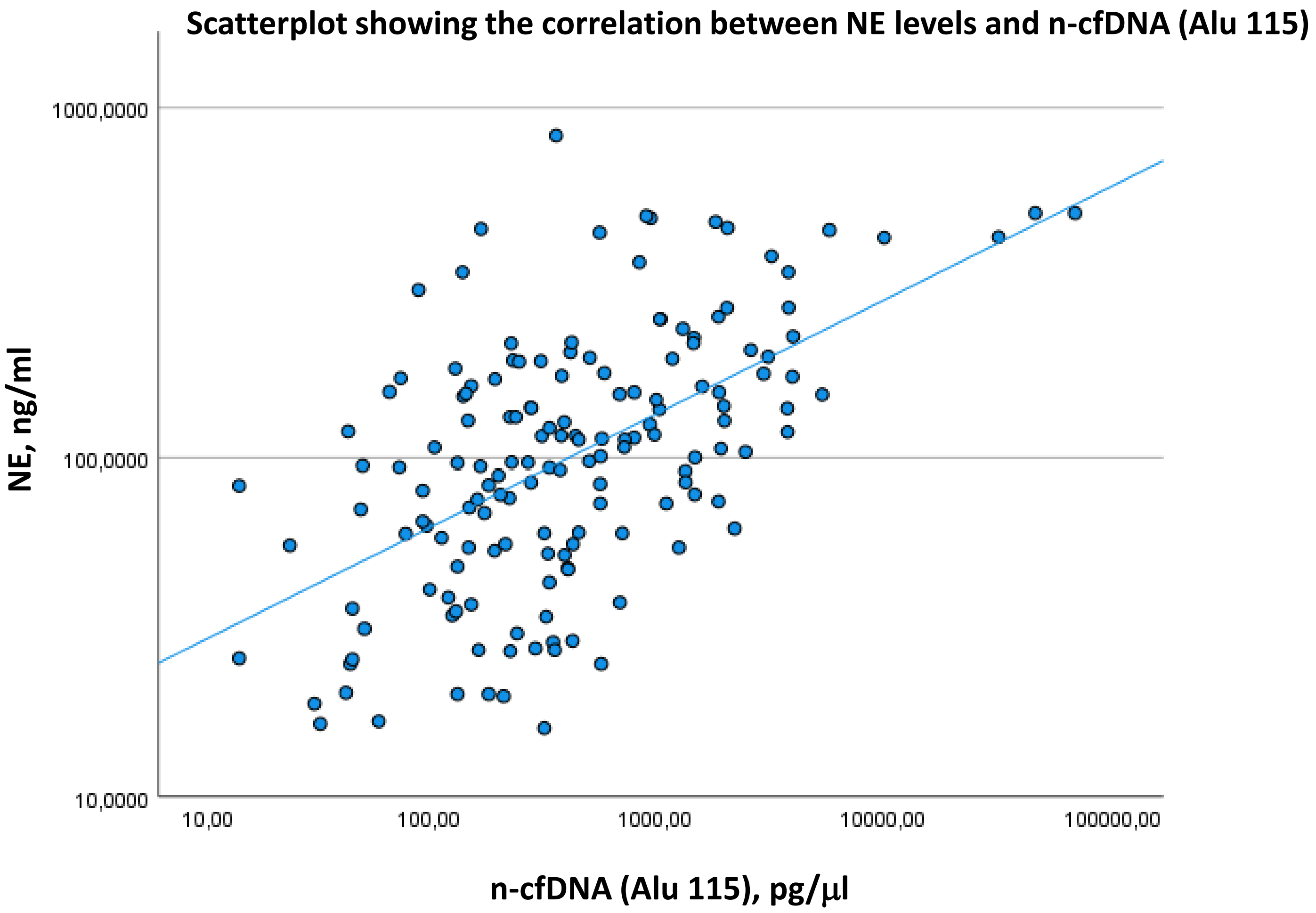

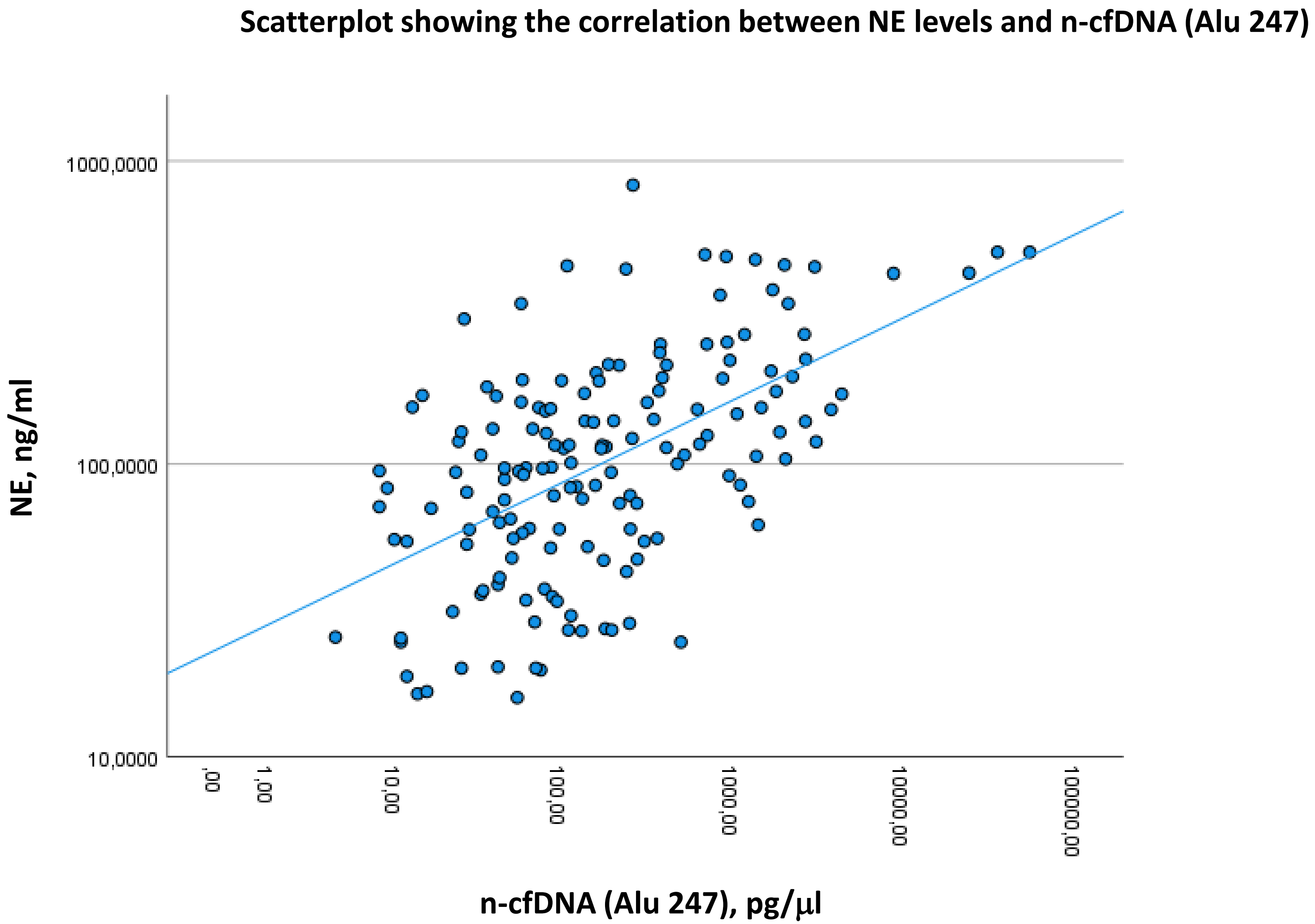
Scatterplot showing the correlation between NE levels and n-cfDNA (Alu 247)
NE, ng/ml
1000,0000
100,0000
10,0000
,00
1,00
10,00
100,00
1000,00
10000,00
100000,00
n-cfDNA (Alu 247), pg/µl

Scatterplot showing the correlation between NE levels and n-cfDNA integrity (Alu 247/115)

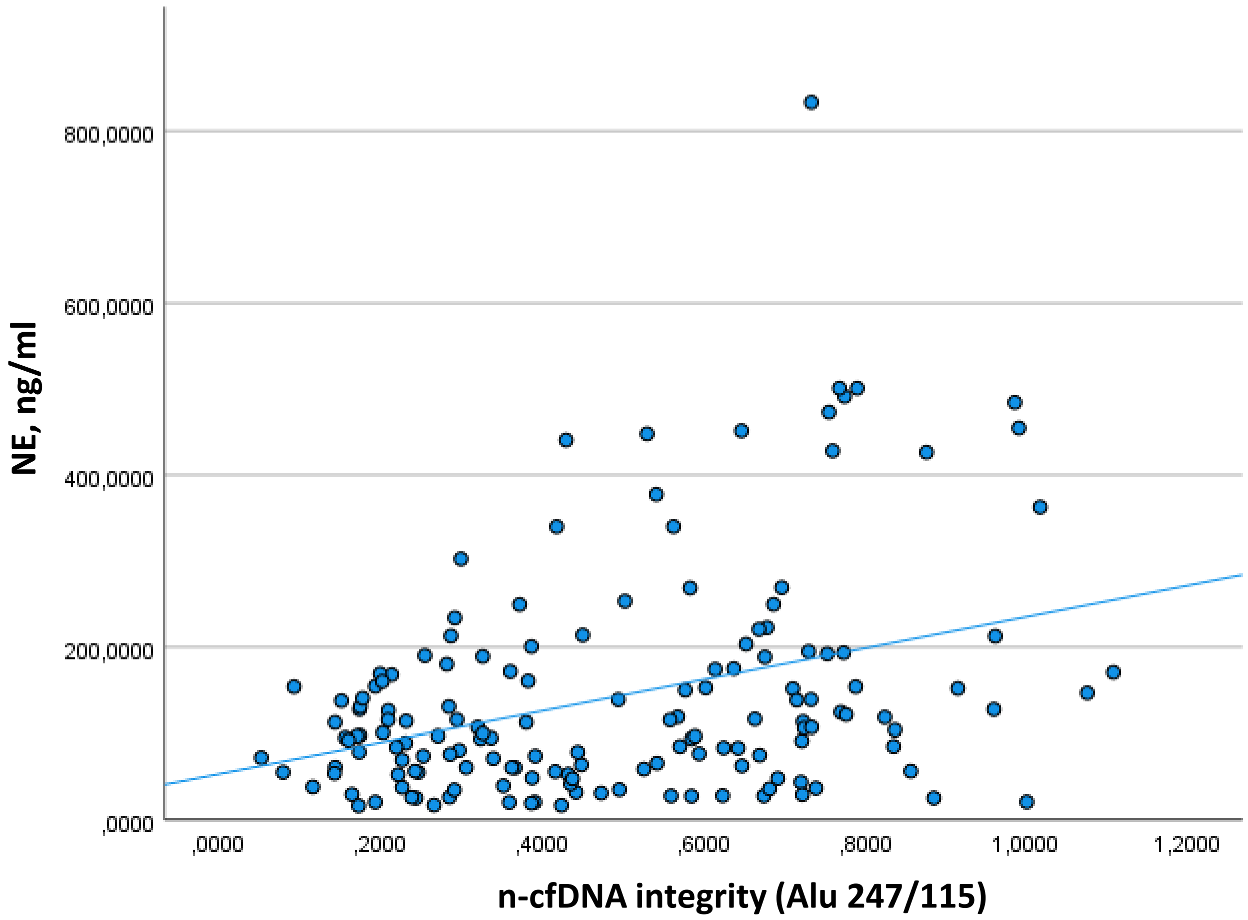

Supplement: Supplementary file 6 — Additional file 6: Supplementary Figures. Scatterplots with the individual data points of the correlation between NE levels and n-cfDNA parameters. [file 12979_2022_315_MOESM6_ESM.pdf]
